# Inactivation of the Nucleus Accumbens Core or Medial Shell Attenuates Reinstatement of Sugar-Seeking Behavior following Sugar Priming or Exposure to Food-Associated Cues

**DOI:** 10.1371/journal.pone.0099301

**Published:** 2014-06-09

**Authors:** Peagan Lin, Wayne E. Pratt

**Affiliations:** Department of Psychology, Wake Forest University, Winston-Salem, North Carolina, United States of America; Texas Christian University, United States of America

## Abstract

Re-exposure to either palatable food or to conditioned stimuli associated with food is known to reinstate food-seeking after periods of abstinence. The nucleus accumbens core and shell are important for reinstatement in both food- and drug-seeking paradigms, although their potential differential roles have been difficult to delineate due to methodological differences in paradigms across laboratories. The present studies assessed the effects of temporary inactivation of the core or shell on priming- and cue-induced reinstatement of food-seeking in identically-trained rats. Inactivation of either the nucleus accumbens core (Experiment 1A; N = 10) or medial shell (Experiment 1B; N = 12) blocked priming-induced reinstatement in an equivalent manner. Similarly, inactivation of the core or medial shell (Experiments 2A & 2B; N = 11 each) also blocked cue-induced reinstatement, although there was also a significant treatment day X brain region X drug order interaction. Specifically, rats with core inactivation reinstated lever-pressing on the vehicle injection day regardless of whether that was their first or second test, whereas rats that had medial shell inactivation on the first day did not significantly reinstate lever-pressing on the second day of testing (when they received vehicle). Yohimbine, while a reportedly robust pharmacological stressor, was ineffective at inducing reinstatement in the current stress-induced reinstatement procedure. These data suggest that both the nucleus accumbens core and shell serve important roles in reinstatement of food-seeking in response to priming and cues.

## Introduction

According to the Centers for Disease Control, over 35% of the adult US population is obese. Obesity-related expenditures were estimated at $147 billion in 2008 and continue to rise with the prevalence of obesity and high cost of health care [1], [2]. Despite efforts that target weight loss by changing dietary habits, most people relapse to their previous eating habits within a few months [3]. Numerous studies suggest that dietary relapse is often influenced by re-exposure to previously avoided foods [4], exposure to cues associated with the avoided foods [5], or exposure to stressful life events [6], [7]. Studies of animal reinstatement of reward-seeking behavior have been increasingly employed as a means to study the neural mechanisms involved in relapse to food-seeking behavior. The reinstatement paradigm is used to model human relapse to drug- or food-seeking [8]–[10] and allows for the potential of independently manipulating the availability of cues predicting food, the availability of the food itself (priming), or the physiological (stress) state of the animal.

There is substantial overlap in the neural systems that engage appetitive behavior directed at food or drug reinforcers. One brain region that is particularly important in guiding motivated behaviors is the nucleus accumbens (NAcc). Pharmacological manipulations of the NAcc affect motivation directed at both appetitive food-seeking as well as consumption if food is freely available (For reviews, see [11]–[14]). Furthermore, early intermediate gene expression within the NAcc is activated by both food- and drug-associated cues [15], [16]. The NAcc is made up of at least two subterritories, the core and the shell, that have clearly distinguishable immunohistochemical and histochemical marker differences, along with different inputs and outputs [17], [18]. The core region, closely aligned with striatal motor pathways, is considered responsible for integrating motivational value and converting these into actions, while the more “limbic” shell, associated with brain regions that process affect-related information, is recognized as integral for processing affective values of environmental stimuli [19]–[21].

How these functional distinctions may apply to food reinstatement in animal models remains to be determined, as these functions for core and shell would both appear to be important components of food-seeking behavior. Relatively few studies have examined the potential differential roles of the NAcc core and shell on the reinstatement of food-seeking in the rat. Notable exceptions include a report by Floresco, McLaughlin, and Hulak [22], in which inactivation of the core region inhibited cue-induced reinstatement while shell inactivation potentiated responding on the previously-reinforced lever. Additionally, Anderson, Schmidt, and Pierce [23] and Yee and colleagues [24] found that blockade of NAcc D2-like dopamine receptors or muscarinic receptors in the shell have little to no effect on the sucrose-prime-induced reinstatement of sucrose-seeking, while blockade of muscarinic receptors in the core do. Thus, there is evidence to suggest a role for the NAcc core in both cue- and priming-induced reinstatement for food-seeking, but perhaps not the shell. This contrasts from prior reports examining the reinstatement of drug-seeking behavior, where blocking dopamine receptors or muscarinic receptors in the shell decreases cocaine-primed reinstatement of drug-seeking, and shell inactivation increases lever-pressing for ethanol [23]–[25]. It is uncertain if these differences occur as a result of the different rewarding properties between natural rewards and drugs of abuse, or whether the specific brain regions and neuromechanisms underlying reward processing is the root of the dissociation. In addition, comparing the role of individual NAcc regions across individual published reinstatement reports has been complicated by the diverse methodology used across laboratories, in which training and reinstatement procedures vary considerably. There has yet to be a systematic examination of the role of NAcc in the reinstatement of food-seeking behaviors in which the training regimen of rats has been consistent across groups of animals while examining the use of different induction methods of reinstatement. Furthermore, there has been no investigation of whether the NAcc core or shell regions may be a final common pathway by which primes, cues, and stress impact motivation to seek out food.

The purpose of these experiments was to examine the functional roles of the NAcc core and medial shell on food-seeking in separate animal groups that were identically trained [22], [26], but in which the induction method of reinstatement differed. Reinstatement was examined following temporary inactivation of either the NAcc core or shell on priming- and cue-induced reinstatement of food-seeking. Here, we report that inactivation of both shell and core attenuated cue- and priming-induced reinstatement. We failed to observe reinstatement following stress induction with yohimbine hydrochloride (at 2 mg/kg); we discuss paradigmatic differences (as compared to previous literature) that may account for this observed lack of effect.

## Methods

All animal care and experimental procedures were run in strict compliance with NIH and Wake Forest University IACUC guidelines for the use of animals in research. The Wake Forest University Institutional Animal Care and Use Committee approved all procedures prior to the implementation of these experiments (Protocol A12–167). All efforts were made to minimize suffering of the animals used in these experiments. At the end of each experiment, euthanasia was accomplished by exsanguination following deep anesthesia with sodium pentobarbital, consistent with AVMA guidelines for the euthanasia of animals.

### Subjects and housing

Seventy-two male Sprague-Dawley rats (Harlan, Madison, WI; *n* = 22–24 for each experiment) were housed in pairs in clear plastic cages in a colony room maintained at ∼21°C with a 12-hr light-dark cycle (lights on/off at 7 A.M./7 P.M.). All behavioral testing was conducted during the light phase. Rats were maintained on food and water *ad libitum* up until 1 wk following surgeries, after which they were gradually reduced to, and maintained at, 90% of their weight by limiting their daily food ration.

### Surgery

Following acclimation to the housing environment, rats underwent cannula placement surgery. They were anesthetized with a Ketamine-Xylazine cocktail (100 mg/kg-10 mg/kg). Using standard aseptic procedures, stainless steel guide cannulas (23 gauge) were implanted bilaterally above the NAcc shell or core. The surgical coordinates, relative to bregma, were: NAcc core (flat skull surgery): 1.3 mm anterior to bregma, M-L: ±1.7 mm, D-V: -5.0; NAcc shell (nose bar at 5 mm above intra-aural plane): 3.1 mm anterior to bregma, M-L: ±1.0; D-V: -5.0 from skull. The guide cannulas were affixed to the skull with screws and dental acrylic, and wire stylets were placed in the cannula to prevent obstruction.

### Apparatus

Six standard operant chambers (Med-Associates, St. Albans, VT, USA) were utilized for the experiments. Each operant chamber was enclosed in a sound-attenuating chamber equipped with a ventilation fan. The chambers were fitted with a houselight and two retractable levers on each side of the central food receptacle. Two identical 100-mA stimulus lights were located above each lever. A programmable speaker, positioned on the wall opposite the food receptacle, presented the auditory stimulus used in these experiments.

### Behavioral Methods

#### Training and extinction

Rats were allowed one week to recover from surgery before gradually being restricted to 90% of their *ad libitum* body weight by limiting their daily ration of rat chow to 8 gr. Rats were maintained at 90% of their *ad libitum* body weight throughout the remainder of the experiment by providing 12–16 g food rations daily, based upon daily monitoring of animal weight. Two days prior to the beginning of magazine training, rats were habituated to the sucrose pellets (45 mg; BioServ) by supplementing their daily food ration with 2 g of the pellets.

The following reinstatement procedure was adapted from the experimental protocol utilized by Floresco et al. [22] and Guy, Choi, and Pratt [26]. Magazine training consisted of two days (Days 1 and 2) in which sucrose pellet delivery was presented randomly for 30 min, with an average of 60 s between deliveries (RT60), in the absence of any levers or stimulus presentations. The day after magazine training was completed (Day 3), both levers were inserted into the chambers for operant training. Rats received 20 min training sessions where lever presses on the active lever resulted in the presentation of a combined CS (light/tone)-UCS (sucrose pellet). Active lever presses were reinforced on a fixed-ratio (FR) 1 schedule in which each lever press resulted in a sucrose pellet primary reinforcer and the presentation of a 5 s light-tone cue (the light above the stimulus lever was illuminated, in conjunction with the presentation of an 80 dB, 3 KHz tone). During the 5 s presentation of the light-tone cue, no additional reinforcers could be earned. As soon as the cue ended, subsequent lever pressing resulted in the presentation of the combined CS-UCS once more and the schedule was repeated. An inactive lever with no programmed consequences was presented on the opposite side of the food receptacle. Left/right positioning of the active lever was counterbalanced across animals in all experimental groups. If a rat did not begin lever-pressing on the first day of FR1, the FR1 training was repeated daily until the rat learned to lever-press for sucrose pellets.

On the next day of training (Day 4 for most animals), rats were presented a fixed-ratio (FR) 2 schedule in which every two lever presses resulted in the same cue-reward presentation. Once again, active lever presses during the cue presentation were recorded, but not reinforced nor counted toward ratio requirements. During days 5–14, rats were shifted to a variable-ratio (VR) 5 reinforcement schedule superimposed upon a fixed-interval (FI) 20 schedule. The VR5-FI20 schedule resulted in the first CS-pellet delivery on an average of 5 active lever presses. After this initial reinforcer was earned, a 20 s time-out period was initiated during which lever presses resulted in no consequences. Following this time-out period, the VR5-FI20 schedule was repeated until the 20 min session was complete. Consistent with prior studies [22], [26], [27], this procedure led to reliably robust responding on the active lever by the end of the operant training period.

Following nine days of VR5-FI20 training, rats underwent daily 20 min extinction sessions in which active and inactive lever presses resulted in no programmed consequences. A rat was considered to have reached extinction criterion (and therefore ready for reinstatement testing) when they had lever pressed less than 10% compared to the last day of VR5-FI20 training on the previously active lever. Individual rats took from 2–6 days to reach this criterion.

#### Reinstatement testing

One day after each rat achieved their extinction criterion, they were subjected to the first of two, 20 min reinstatement sessions separated by a period of 48 h. Across the two test days, rats received, in counterbalanced order, a saline injection and a drug treatment (GABA_A_/GABA_B_ agonist cocktail of muscimol/baclofen, used to inactivate the injected region). A total volume of 0.3 µL was infused; the dose of both drugs was 75 ng/side. This cocktail at this volume and concentration has been shown to be effective for dissociating core/shell functions in prior experiments [22], [28], [29].

For the two groups examining the role of NAcc core and shell on priming-induced reinstatement (Experiment 1A and 1B), rats were placed into the operant chambers, and five non-contingent sugar pellets were delivered one minute prior to the insertion of the levers into the chamber. The levers were then inserted into the chamber for 20 min, during which the rats were able to lever press but not receive either the light/tone cue or any additional sugar pellets (identical to extinction conditions). The number of lever presses on the previously active and inactive levers was recorded in order to assess the amount of incentive motivation the rat had to seek food reward following the priming manipulation.

Two groups of animals were tested to determine the role of the NAcc core and shell on cue-induced reinstatement of food-seeking (Experiments 2A and 2B). The animals in the cue group did not receive non-contingent sugar pellets before the session. The reinstatement sessions consisted of the renewed presentation of the light-tone CS following the first lever press on the previously active lever. Further responding on the previously active lever resulted in the CS presentation on a VR5 schedule, though sugar pellets were never again delivered. There were no time out periods for CS availability on these reinstatement test days.

For experimental groups intended to test the role of NAcc core and shell on stress-induced reinstatement (Experiments 3A and 3B), testing followed an i.p. injection of 2 mg/kg yohimbine hydrochloride (0.5 ml/kg dissolved in sterile water) 25 minutes prior to receiving the intracranial injection of saline or muscimol/baclofen. Once rats had received the NAcc injection, they were immediately placed into the operant chambers for the 20 minute reinstatement test. The animals did not receive non-contingent sugar pellets or any presentations of the light/tone CS during their reinstatement test. Doses of yohimbine were based on previous studies that reported robust reinstatement at this dose [30]–[33].

### Microinfusion procedures

Injections were given through 30-gauge injector cannulas that were inserted 2.5 mm beyond the surgically implanted guides. The injectors were connected with polyethylene tubing to a microdrive pump (Harvard Apparatus, South Natick, MA). Solutions were injected in a volume of 0.3 µL at a rate of 0.3 µL per 45 s, followed by a 1 min rest period in which injectors were left in place to allow drug to diffuse away from the cannula. After drug injections, rats were placed in the operant chambers for reinstatement testing.

To acclimate rats to this procedure prior to experimental testing days, two mock injections were given. Injection procedures were followed as described above, but no solutions were given. On the first mock injection day, injectors were lowered only to the bottom of the guide cannulas. On the second, they were lowered 2.5 mm beyond the guide cannulas into the NAcc. Rats received these two mock injections across the first two days of extinction training, at a disparate time of day, to ensure that the procedures were not predictive of reward unavailability.

### Data Analysis

Dependent measures included the total number of presses on the active and inactive levers. Responding on the sucrose-paired lever (the previously active lever) was compared across the final day of extinction training and both reinstatement sessions. Reward-paired lever responses were analyzed for each experiment using a 2 X 2 X 3 mixed ANOVA with brain region (core or shell), drug order (drug on reinstatement test day 1 or on reinstatement test day 2), and treatment day (last day of extinction, saline reinstatement day, drug reinstatement day). To test whether observed drug effects might be due to non-specific alternations of locomotor activity, total inactive lever presses were also analyzed with a 2 X 2 X 3 mixed ANOVA with the same factors to compare inactive lever responses across the final extinction day and the two reinstatement sessions. Tukey HSD posthoc analyses were conducted to make pairwise comparisons between the last extinction day and the two reinstatement sessions within each experiment, as appropriate. To further examine the patterns of active lever responding within each test session, each of the 20 min sessions were divided into five 4-min epochs and lever presses were graphed across time.

### Histological analysis

**Figure 1:**
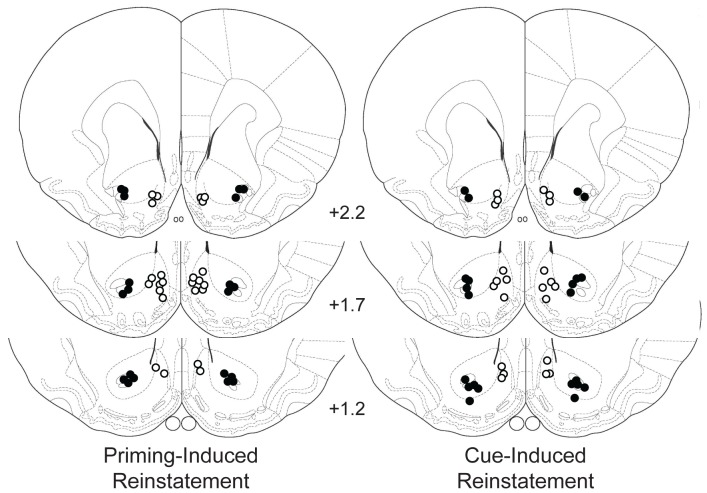
A schematic showing the placement of injector tips for animals included in the analysis following treatment of the NAcc shell (open circles) or core (filled circles) during priming-induced reinstatement (Experiments 1A&B, left panel) or cue-induced reinstatement (Experiments 2A&B, right panel). Numbers represent millimeters anterior to bregma. Drawings were adapted from The Rat Brain in Stereotaxic Coordinates, 4^th^ ed, G. Paxinos and C. Watson, Figures 10, 11, and 13, copyright 1998 [61].

Once experiments were completed, rats were deeply anesthetized with sodium pentobarbital and perfused through the heart with 0.9% NaCl solution followed by 10.0% buffered formalin. Brains were extracted and placed in formalin solution for at least 24 hours. Prior to histological procedures, the brains were transferred to a 10.0% sucrose formalin solution until they sank. Brains were then mounted, frozen, and sliced with a cryostat for histological verification. The 60 µm sections were mounted to glass slides and stained with cresyl violet, and the tips of the cannulas were confirmed by light microscopy to ensure correct injection placement. Figure 1 shows the locations of the injection sites included in analysis. Five rats were excluded from analysis due to cannula placements outside the region of interest.

## Results

### Experiment 1A & 1B: Effect of Nucleus Accumbens Core or Shell Inactivation on Priming-Induced Reinstatement of Food-Seeking

**Figure 2:**
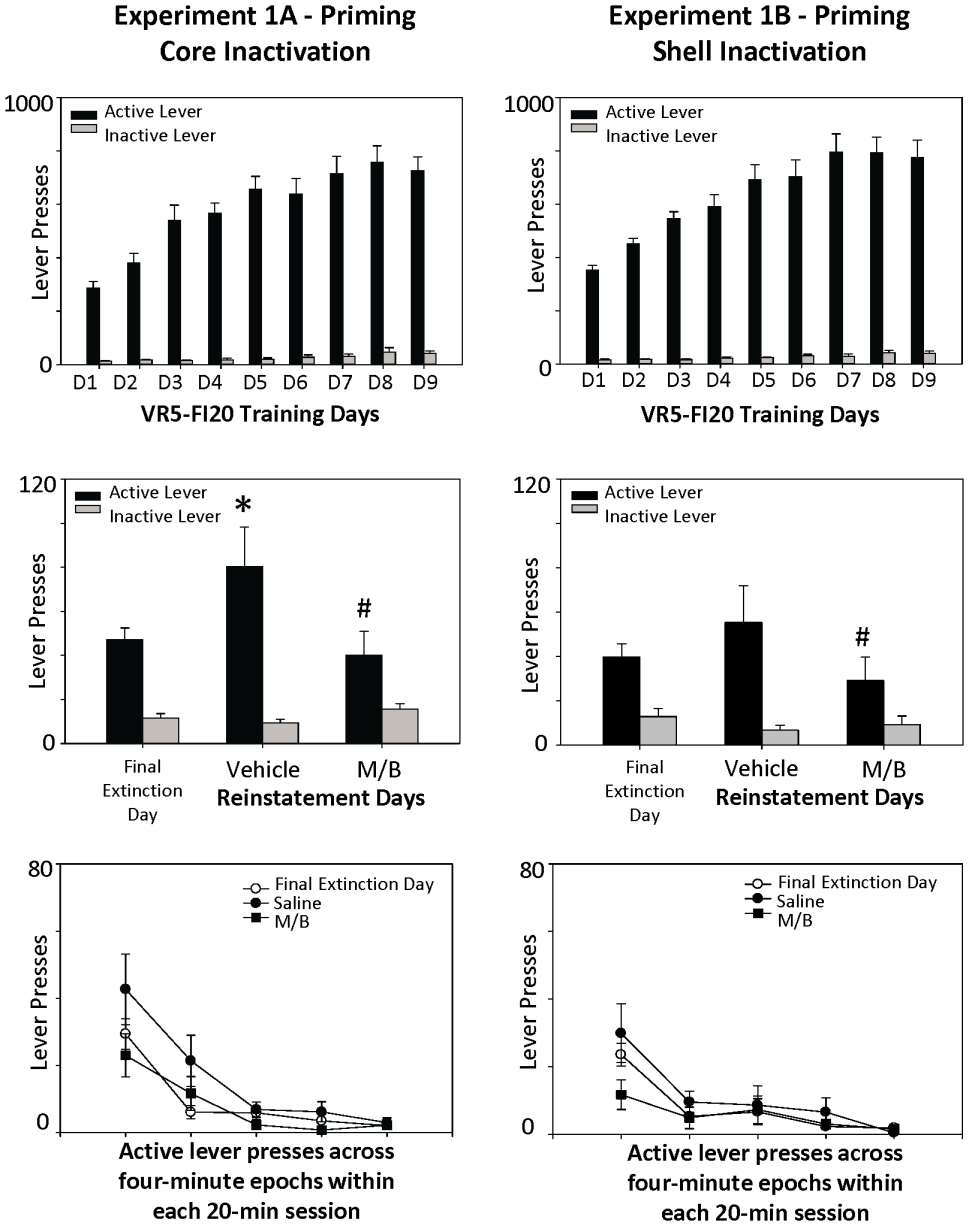
Effects of NAcc core and shell inactivation on priming-induced reinstatement. Top panels demonstrate that rats were showing robust lever-pressing on the VR-5, FI-20 schedule by the completion of training (top panels). The middle panels demonstrate a significant effect of NAcc inactivation on priming-induced reinstatement on the previously active lever, regardless of whether the muscimol/baclofen cocktail was injected into the core or the medial shell. There were no changes in pressing on the inactive lever across extinction and reinstatement test conditions. Bottom panels represent the pattern of lever pressing across the reinstatement period and suggest that drug treatment blocked reinstatement early in the session, which then extinguished across time during all test days. Stars (*) on the bar graphs indicate a significant increase in lever pressing on the saline reinstatement day; number signs (#) indicate a significant decrease in lever pressing activity as compared to that observed on the saline reinstatement day (as determined by Tukey's HSD).

**Figure 3:**
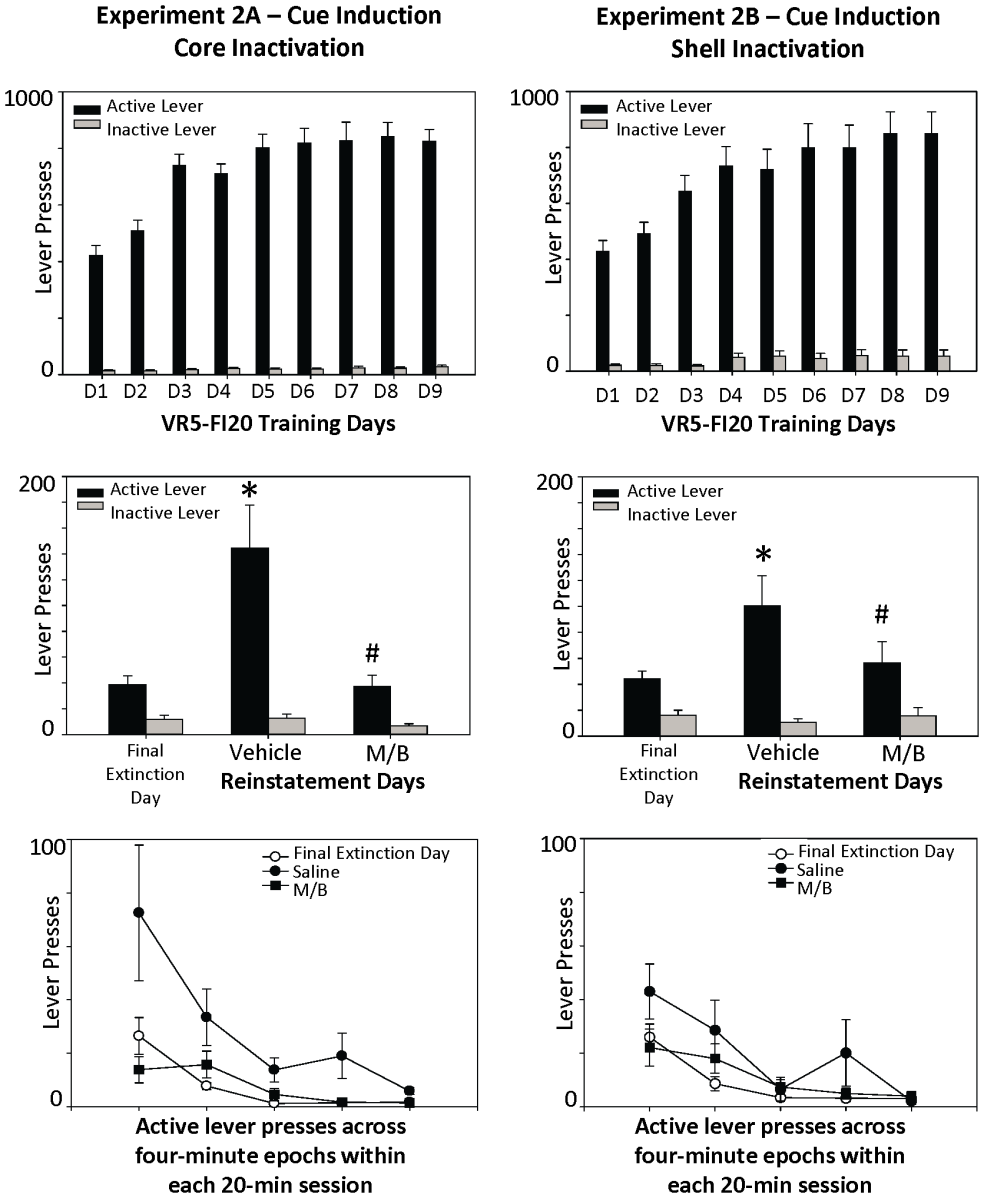
Effects of NAcc core and shell inactivation on cue-induced reinstatement. Top panels demonstrate that rats were showing robust lever-pressing on the VR-5, FI-20 schedule by the completion of training (top panels). The middle panels demonstrate a significant effect of NAcc inactivation on cue-induced reinstatement on the previously active lever, regardless of whether the muscimol/baclofen cocktail was injected into the core or the medial shell. There were no changes in pressing on the inactive lever across extinction and reinstatement test conditions. Bottom panels represent the pattern of lever pressing across the reinstatement period and suggest that drug treatment blocked reinstatement early in the session, which then extinguished across time during all test days. Statistical symbols are consistent with those used in Fig. 2.

During training, all experimental groups lever pressed at higher rates on the active lever relative to the inactive levers, demonstrating that they learned the association between the active lever and the sugar reward (see top panels, Figures 2 and 3). Active lever responding was analyzed with a 2 X 2 X 3 mixed ANOVA, with brain region (core, shell), drug order (whether the drug cocktail was given on the first or second reinstatement day), and treatment day (extinction, saline reinstatement, drug reinstatement) as the independent variables. For Experiment 1, (total *n* = 22), inactivation of the NAcc core or shell effectively suppressed the reinstatement of food-seeking when induced with a prime (Figure 2, middle panels). The analyses indicated a significant main effect of treatment day on active lever pressing behavior, *F*(2, 36) = 3.93, *p* = .03, such that inactivation of either the NAcc core or shell blocked priming-induced reinstatement. There was no a significant interaction effect of treatment day x brain region for active lever responding, *F*(2, 36) = .21, *p* = .81, indicating that inactivation of the core and shell had equivalent effects on priming-induced reinstatement. There was no interaction effect of drug order x treatment day, *F*(2, 36) = .14, *p* = .87, nor of treatment x brain region x drug order *F*(2, 36) = 1.22, *p* = .31. As shown in the bottom panels of Figure 2, reinstatement occurred early in the session, after which animals ceased to respond by the end of the 20 minute test session. Lever pressing on the inactive lever did not significantly differ across the final extinction day and the two reinstatement tests (all p's>0.05).

### Experiment 2A & 2B: Effect of Nucleus Accumbens Core or Shell Inactivation on Cue-Induced Reinstatement of Food-Seeking

Inactivation of either the NAcc core or shell also blocked reinstatement of food-seeking when induced with cues (Figure 3, middle panels). The mixed ANOVA yielded a significant main effect of treatment day on active lever pressing, *F*(2, 36) = 12.35, *p*<.001, such that injections of the muscimol/baclofen drug into either the NAcc core or shell attenuated cue-induced reinstatement. For both core and shell inactivation, inhibition of reinstatement occurred early in the session; all animals ceased responding by the end of the 20 minute test session (Figure 3, bottom panels).

**Figure 4:**
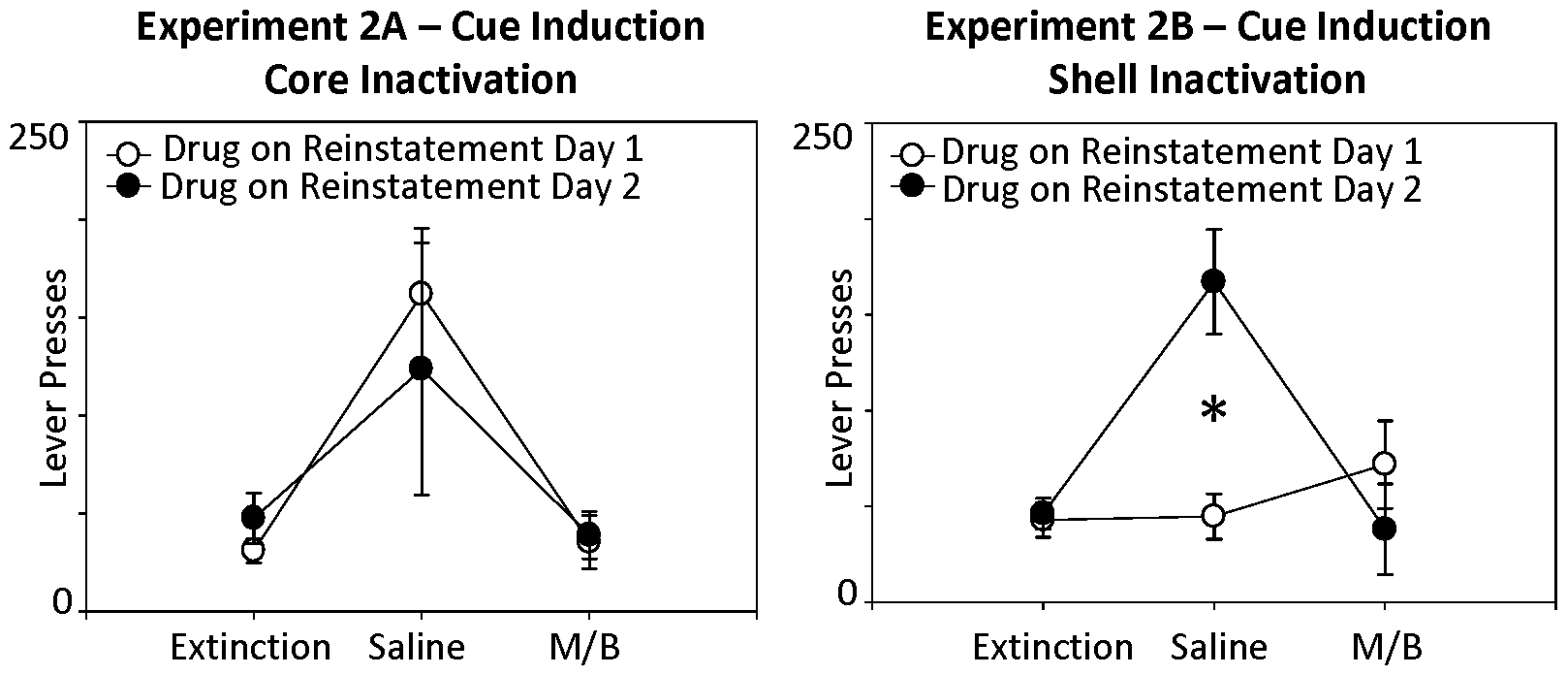
Although NAcc core and shell inactivation caused a main effect of drug on cue-induced reinstatement (Fig. 3), there was also a significant three-way interaction. As shown here, the rats who received muscimol/baclofen injections in the NAcc shell on the first reinstatement day did not reinstate food-seeking behavior on the second test when they received saline injections. The star represents a significant difference in responding between animals that received saline injections on the first or second test day. In contrast, rats with NAcc core inhibition showed attenuated reinstatement regardless of which day the drug was given.

There was no significant interaction effects of treatment day x brain region [*F*(2, 36) = .59, *p* = .56] or treatment day X drug order [*F*(2, 36) = .49, *p* = .62], indicating that inactivation of the core and shell had equivalent effects on cue-induced reinstatement and that, in general, the injection of the baclofen/muscimol blocked cue-induced reinstatement in both brain regions. However, there was a significant three-way interaction of treatment day x brain region x drug order, *F*(2, 36) = 8.83, *p* = .001. As shown in Figure 4, this appears this was due to the fact that animals who received the drug cocktail into the NAcc shell on the first reinstatement test day did not subsequently reinstate responding on the saline test day (second reinstatement test day). No significant effects were found across the three treatment days on inactive lever pressing behavior (all *p*'s>.05).

### Experiment 3A & 3B: Treatment With Yohimbine Failed to Elicit Stress-Induced Reinstatement in the Absence of Priming or Conditioned Cues

**Figure 5:**
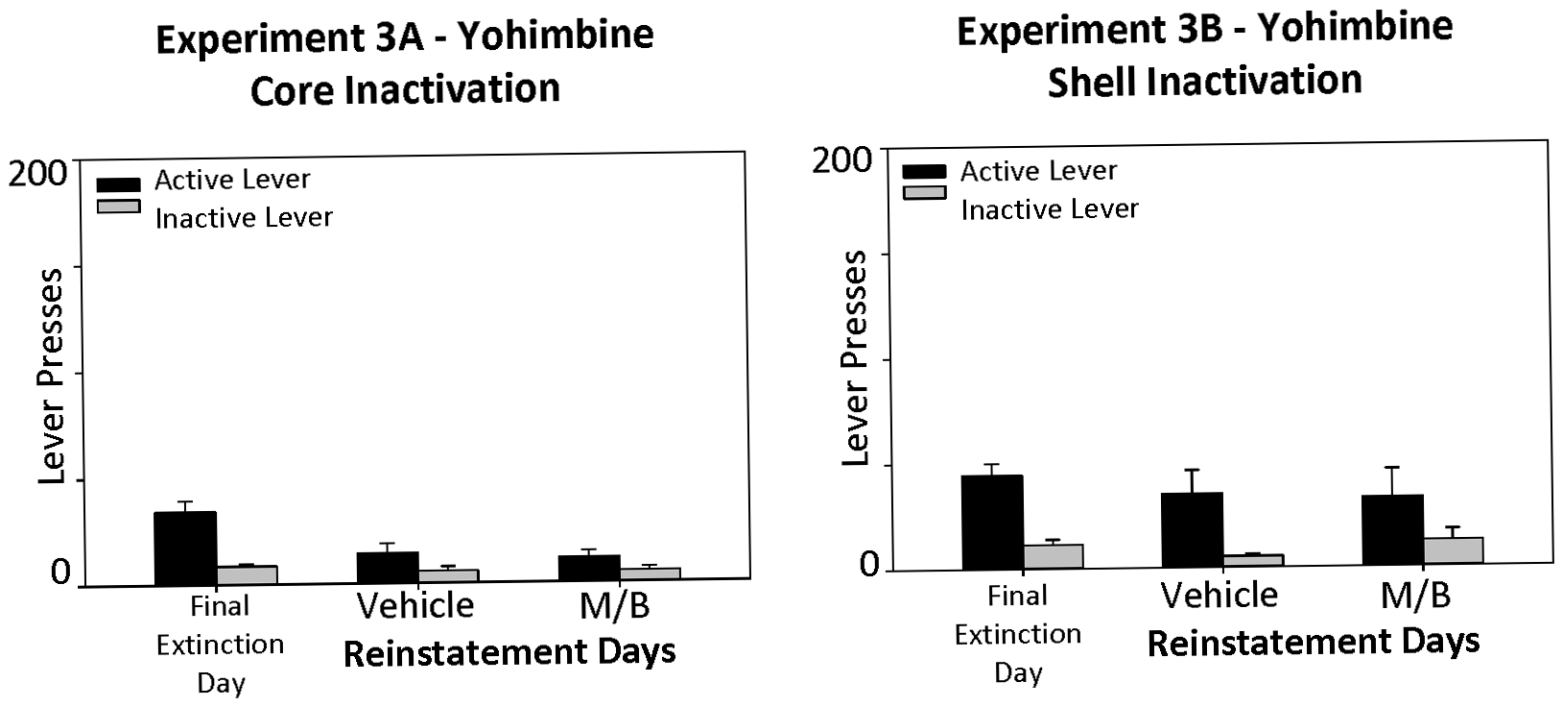
Rats tested for reinstatement following injections of the pharmacological stressor yohimbine did not reinstate food-seeking behavior. As can be seen, there was no increase in lever pressing following yohimbine, even on the day that rats were tested with saline vehicle injections into either the NAcc shell or core.

Reinstatement of food-seeking behavior induced by the pharmacological stressor yohimbine was not observed in Experiment 3 (total *n* = 23) (Figure 5). There was no difference in active lever pressing between extinction and reinstatement test days, *F*(2, 38) = 2.49, *p* = .10, across groups with core or shell injections. Furthermore there was no effect of inactivation of either the core or shell on active lever-pressing behavior across treatment days [treatment day x brain region: *F*(2, 38) = .35, *p* = .71]. There was no effect of drug injection order on active lever pressing behavior, [treatment day x drug order: *F*(2, 38) = .20, *p* = .82], nor a treatment day x brain region x drug order effect, *F*(2, 38) = .36, *p* = .70. Inactive lever presses were equivalent across all testing days and drug conditions (all *p*>.05).

## Discussion

The present experiments tested the effects of nucleus accumbens core or shell inactivation on priming-, cue-, and stress-induced reinstatement of food-seeking. Our goal was threefold: to investigate whether the NAcc is potentially a final common pathway in all three food-directed reinstatement paradigms, to determine if there is a distinction between the NAcc core and shell's role in the differing types of reinstatement, and to address both questions while utilizing identical methodology during training and extinction across all groups. In these experiments, we found that inactivation of the core or shell attenuated both priming- and cue-induced reinstatement of food-seeking. We were unable to assess the potential role of the NAcc on stress-induced reinstatement because, contrary to prior stress-induced reinstatement studies using yohimbine as a pharmacological stressor [8], [10], [30], [32], yohimbine did not induce reinstatement in the present experiments.

In contrast to the effect observed on the formerly active lever, drug treatment did not alter lever presses on the formerly inactive lever. This suggests that the effects observed were due to a specific reduction in reinstatement, as opposed to general sedative effects secondary to drug treatment. In these experiments, we did not test whether NAcc core or shell inactivation impacted lever-pressing during ongoing reinforced responding, which would have provided additional evidence that the observed effects were specific to reinstatement *per se* [34]. However, in addition to the lack of effect on the inactive lever across all experiments, neither core nor shell inactivation reduced lever pressing on the formerly active lever for the rats treated with yohimbine. Though the yohimbine-treated rats did not significantly reinstate following injections of the pharmacological stressor, the baseline pressing on the active lever was two to three times higher than activity on the inactive lever, and sufficient to permit detection of gross inhibition of responding due to inactivation of these brain regions had it occurred here. Finally, prior studies that have examined the effects of muscimol/baclofen treatment of the nucleus accumbens have shown either no effect [35] or transient effects [36] on locomotor activity in rats. Thus, it is unlikely that the impact of inactivation on priming- and cue-induced reinstatement, discussed further below, was due to gross locomotor inhibition or sedation due to the regional inactivation.

### Effects of NAcc Core or Shell Inactivation on Priming-Induced Reinstatement of Food-Seeking Behavior

The current experiments suggest that both the core and shell play a significant role in priming-induced reinstatement of food-seeking. Lever pressing on the previously-reinforced lever was reduced following either NAcc core or shell inactivation with the GABA_A_/GABA_B_ agonist cocktail, and there was no difference in this attenuation across the core/shell groups. Previously, few studies have systematically examined the differential contributions of the core and shell in priming-induced reinstatement, and studies that have done so primarily focused on drug-priming-induced relapse rather than food. For example, McFarland and Kalivas [35] reported that muscimol/baclofen inactivation of the core attenuated cocaine-primed reinstatement of cocaine-seeking, consistent with the current experiment's finding of attenuation in food-primed reinstatement. Furthermore, they reported that inactivation of the shell had no effect on cocaine-primed reinstatement. Other researchers [23], [24] have reported that selective receptor antagonism (of dopamine and muscarinic acetylcholine receptors) in the shell had no effect on sucrose-primed reinstatement of sucrose-seeking but decreased cocaine-primed reinstatement of cocaine-seeking, while the same manipulation applied to the core attenuated both sucrose- and cocaine-primed reinstatement. Regarding these findings, it is important to note that selective receptor antagonism is not equivalent to muscimol/baclofen inactivation, as done here [23], [24]. Nonetheless, in combination with previous experiments, these data suggest that the functional integrity of the mesolimbic dopaminergic system (including the NAcc) is indispensable for priming-induced reinstatement, regardless of whether the reward is a drug or food.

McFarland and Kalivas [35] also assessed whether inactivation of the core affected food-primed reinstatement and reported that inactivation of the core did not reduce food-seeking. These authors did not examine the potential role for the NAcc shell. Nonetheless, the contrast with the current data may be attributable to differences in how the rats were primed between experiments. In the present study, five sugar pellets were dropped into the hopper in the minute prior to the reinstatement sessions, whereas McFarland and Kalivas placed one food pellet in the hopper before beginning the test session and then dropped five more pellets at 2 minute intervals during the first ten minutes of the reinstatement session. This is an important difference because the rats in the current experiments were not able to lever press while they were consuming the pellets, as the levers had not yet been inserted into the chamber. In the McFarland and Kalivas experiment, it is possible that ongoing lever-pressing may have been followed by a sugar pellet delivered on the 2 minute delivery cycle, which may have additionally renewed the response-outcome association.

At present, this experiment is the first to directly compare the effect of NAcc core and shell inactivation on priming-induced reinstatement of food-seeking. These findings suggest that the core and shell are both fundamental for mediating the expression of priming-induced reinstatement to food-seeking.

### Effects of NAcc Core or Shell Inactivation on Cue-Induced Reinstatement of Food-Seeking Behavior

In these experiments, pharmacological inhibition of either the core or shell inhibited cue-induced reinstatement of food-seeking. That inactivation of the NAcc core blocked reinstatement of food-seeking is consistent with a number of prior studies [22], [36]–[40]. However, our finding that inactivation of the shell also blocked reinstatement in this cue-induced paradigm adds to an already inconsistent literature. Specifically, Floresco et al. [22] reported enhanced reinstatement after inactivation of the NAcc shell in a reinstatement paradigm similar to the one used here, but others have reported no effect or an attenuated effect in cue-induced reinstatement of food- or drug-seeking following inactivation or dopaminergic receptor antagonism in the shell [36], [41].

Although the current data contrast with that seen by Floresco and colleagues, they are not inconsistent with recent studies examining the ability of a Pavlovian conditioned cue to invigorate instrumental responding in extinction conditions. For example, it was found that inactivation of the NAcc shell abolished the outcome-specific Pavlovian Instrumental Transfer (PIT) effect [42]. Pavlovian incentive learning mediates expectancy of a reward associated with a conditioned stimulus, and it is thought that this expectancy state motivates operant behavior [43]. A Pavlovian reward-associated stimulus presented after operant extinction training can reinvigorate responding for at least two possible reasons. First, the cue itself may increase the expectancy of the reward that has been associated with it, increasing the motivation to perform the operant response. Second, the stimulus itself may serve as a secondary reinforcer and thereby strengthen the operant response. Corbit and Balleine [42] found that the shell is more important for the former. Applied to cue-induced reinstatement studies, the Pavlovian stimuli of the light-tone cue may therefore produce an expectancy of the sugar pellet reward and thus potentiate the operant lever-pressing response due to its incentive value. If PIT is an important function of the shell, as suggested by Corbit and Balleine, then the current data are consistent with recent reports of the accumbens core and shell's roles in such PIT tasks, as shell inactivation apparently blocks the ability of reward-associated cues to invigorate operant responding.

Additionally, it should be noted that there was a significant interaction effect of treatment day x brain region x drug order. The animals in the core group that received drug on the first testing day reinstated lever-pressing behavior when tested 48 hours later with saline infusions. However, the animals in the shell group that received drug on the first reinstatement day of testing did not reinstate the operant behavior when subsequently tested with vehicle infusions (Figure 4). To the extent that these data reflect a possible core-shell distinction in function, this suggests that the core might be vital for updating the incentive value of the light-tone cue during extinction. Rats with shell inactivation may still have been able to update this representation for the second day of testing (thus not reinstating on the second day), whereas inactivation of the core may have blocked the ability of the rats to learn extinction to the light-tone cue, and so the animals in this group generally showed reinstatement when injected with saline regardless of whether it was the first or second reinstatement test day. Though further research is necessary to validate this potential function of the core in rigorous Pavlovian extinction models utilizing cues predictive of food, this interpretation is consistent with recent reports that suggest a critical role for the NAcc core in regulating extinction to cocaine-paired cues [44], [45]. Furthermore, the NAcc core is essential for modulating conditioned preparatory processes involved in Pavlovian associative learning, and selective core lesions can impair discriminatory approaches to a Pavlovian conditioned stimulus [46].

Together, the data show a critical role for the NAcc core and shell in cue-induced reinstatement, as inactivation of these regions attenuated the reinstatement effect to food-associated cues. Additionally, the interaction effect suggests that the two regions may have differential effects on cue-induced reinstatement of food-seeking, perhaps in terms of updating the value of incentive stimuli.

### Yohimbine as a Pharmacological Stressor in the Current Experiment

Yohimbine was ineffective in inducing reinstatement responding in this study. The fact that yohimbine did not have the intended effect was surprising, as the drug has been successfully used as a pharmacological stressor in numerous stress-induced reinstatement of drug- and food-seeking studies at the same dose utilized here [30], [47]–[52]. In light of those reports, the current literature on the effects of yohimbine on operant performance is mixed. Studies that have used a similar dosage of yohimbine have reported an increase [53], no effect [54], or a decrease [55] in operant lever-pressing response maintained by food. We did not perform any anxiety measures on the rats post-injection, but most rats injected with yohimbine showed piloerection of the fur and appeared to decrease their locomotor activity in their home cage in a manner consistent with behavioral effects observed in other studies [56], [57]. Therefore, it appeared that the yohimbine treatment was behaviorally effective, but that it nonetheless did not reinstate operant responding.

It may be that the failure to observe yohimbine-induced reinstatement in this report resulted from substantial methodological differences across laboratories. For example, the current study employed 20 minute training sessions for nine days. This is a stark contrast to other stress-induced reinstatement of food-seeking reports, where rats underwent training sessions ranging from three [32] to nine hours daily [30], or during overnight sessions followed by short daytime access for 10–30 days [58]. The longer daily training sessions given across an extended period of time may certainly have strengthened the association between the operant response and reward, but the shorter sessions utilized here decreased the likelihood that the rats would become sated during the training sessions [22]. It is also possible that the shorter, 20 minute reinstatement test session was not long enough to measure yohimbine effects on reinstatement, as yohimbine has been reported to have a half-life of up to eight hours in the rat [59]. However, Nair and colleagues [32] showed that following yohimbine injections, reinstatement responding can be observed within the first 30 minutes of testing, after which responding appears stable for the next 60 minutes. We chose the shorter time frame for these experiments to be consistent in the reinstatement methodology across all three reinstatement paradigms tested here (priming, cues, and stress), and also to reduce concerns about potential diffusion of the muscimol/baclofen cocktail across the shell/core border or into other brain regions during the course of the testing.

Another methodological difference between the stress-induced reinstatement as run here and in prior studies is the absence or presence of the light-tone cue across extinction and reinstatement sessions. To be certain that the reinstatement was only elicited by the yohimbine itself, these experiments did not include presentation of the light-tone cues during extinction or reinstatement testing. In other yohimbine-induced reinstatement of food-seeking studies [30], [32], [58], active lever pressing continued to result in the presentation of light-tone cues during extinction and reinstatement, though no food was delivered. Despite these efforts to extinguish the association between pellet delivery and the light-tone cues during extinction training and render the reward-paired stimuli “neutral”, we speculate that the presentation of the cues during reinstatement testing may be vital for producing reinstatement. Similar to present results, Shelton and Beardsley [60] reported that footshock (a stressor) did not induce reinstatement of lever-pressing behavior when the light-tone CS that was present during cocaine self-administration was omitted during extinction training and reinstatement testing. In contrast, animals that were presented with the light-tone CS during extinction and footshock-induced reinstatement sessions showed significant reinstatement of cocaine-seeking behavior. Paired with the current findings, this suggests that stress may induce reinstatement by enhancing the incentive value of reward-associated cues, instead of directly increasing the motivation to seek the reinforcer *per se*.

### Concluding Remarks

The present findings suggest that the NAcc is an important component of the neural circuitry through which re-exposure to palatable food and palatable food-associated stimuli can influence motivation and food-seeking behavior after a period of abstinence. This study demonstrates that under these conditions, priming- and cue-induced reinstatement are blocked when either the NAcc core or shell are inactivated, and suggest that the core and shell may have dissociable roles in regulating cue-induced reinstatement of food-seeking, such that the core may be vital to learning about stimuli devaluation. These results have implications for the understanding of the neurocircuitry of food-prime- and food-cue-induced relapse to previous unhealthy eating habits, and suggest that modulating NAcc core and shell activity might be able to attenuate palatable food craving and relapse in humans that are dieting. With the continual and expected rise of obesity, future research aimed towards developing a clearer understanding of the neural circuitry regulating reinstatement will be beneficial in determining pharmacological strategies to help with dietary relapse.
